# FLMMIF: privacy-preserving federated multi-modal medical image fusion

**DOI:** 10.3389/frai.2026.1857709

**Published:** 2026-07-14

**Authors:** Lei Meng, Shuangsong Ren, Jing Wang, Ying Che

**Affiliations:** 1 Department of Ultrasound, The First Affiliated Hospital of Dalian Medical University, Dalian, Liaoning, China; 2 Department of Medical Ultrasound Health Medical Department, Central Hospital of Dalian University of Technology, Dalian, Liaoning, China

**Keywords:** federated learning, image fusion, low-rank, medical image, multi-modal image, privacy-preserving

## Abstract

As a pivotal technique in smart healthcare, medical image fusion integrates complementary functional and structural information to facilitate accurate diagnosis and enhance clinical decision-making reliability. However, existing centralized methods typically raise serious data privacy concerns, while standard distributed approaches often fail to balance global generalization with local node personalization due to data heterogeneity. To address this, we propose FLMMIF, a privacy-preserving framework integrating a federated learning paradigm and low-rank adaptation for personalized and secure medical image fusion. During the local training phase, we utilize a dual-branch encoder and single-branch decoder, adopting a two-stage iterative strategy: initially training low-rank parameters to secure local personalization, followed by training full-rank parts to guarantee global baseline performance. Subsequently, this iterative process ensures that the model dynamically coordinates specific local features with general global knowledge before parameter transmission. Finally, we establish a metric-based aggregation mechanism on the server, FedIF, which evaluates the performance of uploaded models to assign higher aggregation weights to superior nodes for optimized global updating. Experimental results demonstrate that FLMMIF generates high-quality fusion results that effectively protect data privacy while achieving precise node-specific personalization.

## 1 Introduction

Medical imaging plays a pivotal role in clinical settings, enabling the analysis of patient anatomical structures and pathological changes based on specific imaging physics (Hendee and Ritenour, 2003; Bushberg and Boone, 2011). For instance, computed tomography (CT) excels at visualizing dense tissues such as bones and calcifications, while magnetic resonance imaging (MRI) offers superior contrast for soft tissues and organs. However, a single imaging modality is often insufficient to comprehensively characterize a patient's condition, lacking complementary information such as functional metabolism or precise spatial localization. Medical image fusion addresses this limitation by integrating salient features from multiple source images into a single fused representation. This technique has been widely adopted in medical diagnosis and treatment planning; for example, CT/MRI fusion is critical for precise positioning in radiotherapy and neurosurgery, while PET/MRI fusion combines metabolic activity with anatomical context, making it highly suitable for early tumor detection and functional brain imaging.

**Figure 1:**
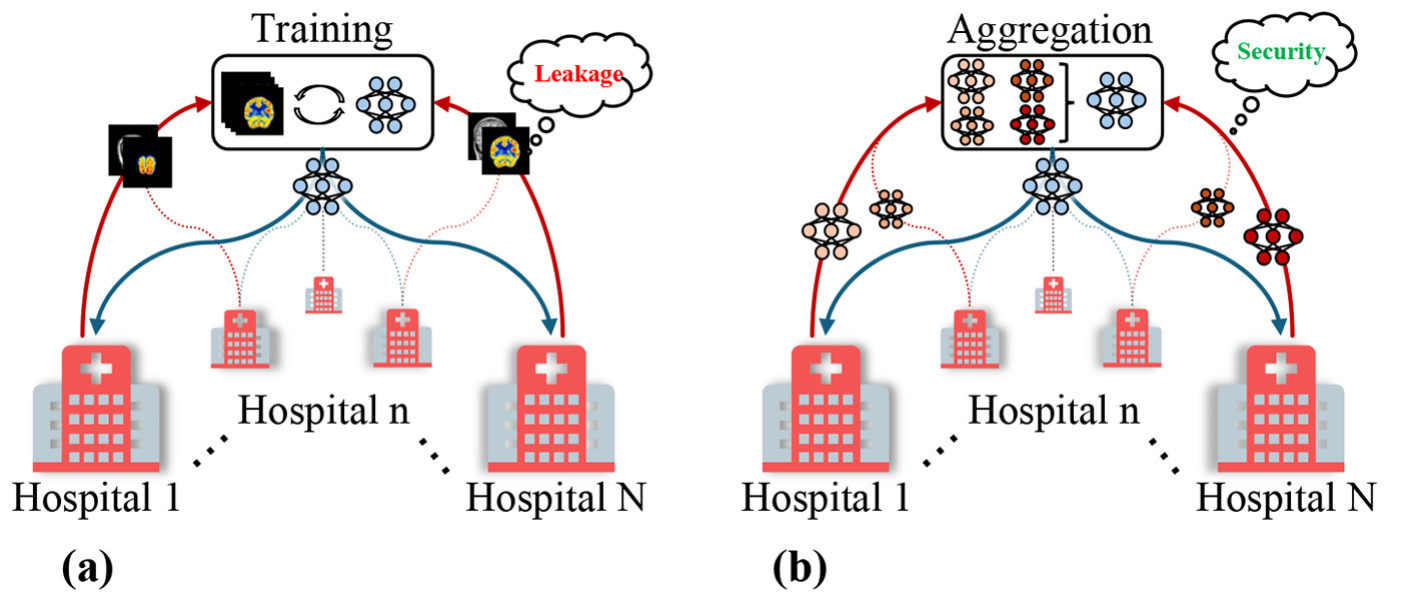
The motivation of our method. **(a)** Existing fusion methods. **(b)** Our federated fusion method.

Despite remarkable progress, existing fusion methods are typically constrained by the requirement of centralized data collection, resulting in distinct “data silos” due to privacy regulations. As illustrated in Figure 1, most current approaches necessitate aggregating training data from various institutions into a central server, failing to comply with strict data privacy laws (e.g., HIPAA, GDPR) (Truong et al., 2021). Consequently, institutions cannot freely share sensitive patient data to train high-performance fusion models. Therefore, designing a privacy-preserving medical image fusion method capable of training on distributed data while maintaining model performance and ensuring data security would significantly enhance the accessibility and utility of computer-aided diagnosis.

Furthermore, current distributed methods often suffer from statistical heterogeneity, resulting in a global model that generalizes poorly to local distributions (Chung et al., 2026; Sun et al., 2025). Clinical centers, such as hospitals using different scanner vendors, typically possess data with unique feature distributions that a single global model cannot perfectly accommodate. Therefore, there is an urgent need to establish an efficient personalization mechanism—such as parameter-efficient fine-tuning—that bridges the gap between general global knowledge and specific local characteristics, enabling local nodes to maintain high performance on their private data.

In recent years, federated learning (FL) has emerged as a dominant technology in privacy-preserving computing, capable of collaboratively training models without sharing raw data (Zhang et al., 2021; Mammen, 2021). However, in the field of medical image fusion, applying standard FL is challenging due to the conflict between diverse local features and a unified global objective. Consequently, existing FL-based fusion methods struggle to balance the trade-off between global generalization and local personalization. Thus, a key research question remains: how to leverage the collaborative power of federated learning to force the network to learn shared representations while preserving unique local capabilities, thereby increasing their clinical utility across different institutions.

Low-rank adaptation (LoRA) (Hu et al., 2022) offers a potential solution by freezing pre-trained weights and injecting trainable rank decomposition matrices to reduce computational overhead. In the context of federated medical image fusion, a major challenge lies in the severe intra-modality distribution shifts caused by distinct scanner vendors and clinical protocols across different institutions. Existing federated medical image fusion models require a delicate balance between feature extraction and reconstruction capabilities, making them unsuitable for simple low-rank injection without structural adaptation. Therefore, we introduce LoRA as a structural decoupling mechanism explicitly designed for multi-modal medical image fusion. By explicitly isolating personalized, scanner-specific representations within the low-rank matrices and maintaining generalizable fusion knowledge in the shared full-rank parameters, our framework can effectively mitigate intra-modality distribution shifts. Adapting LoRA techniques to iteratively decouple and train the personalized and global parameters of a specialized fusion network represents a promising direction to enhance diagnostic flexibility.

To address these challenges, we propose a privacy-preserving multimodal medical image fusion method, named FLMMIF. This framework generates robust fusion results from heterogeneous distributed data and supports effective domain adaptation for specific modalities across different clients. Specifically, to prevent inter-modality interference and preserve distinct anatomical or functional information, we utilize a dual-branch encoder architecture where each branch independently extracts features from a specific source modality. Then, we embed LoRA modules within these independent branches to specifically mitigate intra-modality distribution shifts across clients, ensuring that the model adapts to local data variations without compromising the backbone capabilities. Subsequently, during the training phase, we employ a two-stage iterative strategy that alternately optimizes the low-rank parameters to capture personalized local distributions and the full-rank parameters to maintain global consistency. Finally, during the aggregation phase, we implement FedIF mechanism to integrate these updates, yielding fusion results that are robust to data heterogeneity and precisely aligned with clinical standards across different institutions.

## 2 Related work

This section briefly reviews recent advancements in multi-modal medical image fusion, privacy-preserving medical image processing, and federated learning.

### 2.1 Medical image fusion

Medical image fusion aims to combine the advantages of multi-modal medical images to generate a fused image with more comprehensive information, and it has been widely applied in fields such as computer-aided diagnosis (James and Dasarathy, 2014). Li et al. (2025) propose a bidirectional stepwise feature alignment and fusion method. By introducing a modality discrepancy-free feature representation, this method effectively reduces the negative impact of modality differences during cross-modal feature matching, and achieves the alignment and fusion of unaligned medical images within a unified single-stage framework. Zhou et al. (2024) propose utilizing cross-modal learning and information enhancement techniques, combined with multi-module feature processing and a customized loss function, to achieve the effective fusion of multimodal medical images. Fan et al. (2025) propose a lightweight fusion network combining discrete wavelet transform and the Mamba architecture to effectively extract modality-specific frequency domain features, balance local details with global semantics, and improve the fusion quality and robustness of multimodal medical images. Zhao et al. (2023) propose a multi-discriminator hierarchical wavelet generative adversarial network that improves the fusion quality of multimodal images by extracting multi-scale and edge information through a hierarchical wavelet fusion module and an edge perception module. However, existing fusion methods require the collection of multimodal medical data from users, making them vulnerable to privacy leakage.

### 2.2 Privacy-preserving medical image processing

The privacy of medical images is of paramount importance, and existing image processing methods have already achieved user privacy protection in areas such as image detection (Yang et al., 2022; Liu et al., 2022), segmentation (Pietrantoni et al., 2023), and processing (Wu et al., 2024, 2025). Yang et al. (2022) propose a dynamic region-aware graph convolutional network that utilizes a self-attention mechanism and a graph convolutional network to adaptively model the interactions between regions, thereby improving the detection accuracy of privacy-leaking images. Liu et al. (2022) propose a lightweight privacy-preserving Faster R-CNN framework that combines additive secret sharing with edge computing, and improves secure computation sub-protocols to achieve efficient privacy-preserving computation. Pietrantoni et al. (2023) propose a privacy-preserving visual localization framework based on image segmentation. By jointly learning global and local features to construct compact 3D maps, this approach prevents the reconstruction of personal privacy information from the original images. Barut et al. (2022) propose a residual 1-D image Transformer model that significantly improves the classification accuracy of multi-class malicious traffic and cross-protocol generalization while protecting data privacy by extracting network header byte sequences without parsing sensitive payloads and IP ports. Qi et al. (2022) propose a privacy-preserving image classification method combining encrypted images with the Vision Transformer, which utilizes ViT's patch embedding and position embedding features to effectively reduce the impact of block-wise image transformation while maintaining classification accuracy and robustness against various attacks. However, there is currently a lack of data protection methods directly targeting multimodal medical image fusion.

### 2.3 Federated learning

#### 2.3.1 General federated learning

Google proposes federated learning (FL) to address the severe challenges of data privacy leakage and isolated data silos in traditional centralized machine learning (Kairouz and McMahan, 2021). By enabling multiple clients to collaboratively train a shared global model without exchanging raw sensitive data, this decentralized paradigm inherently protects user information. As the fundamental aggregation algorithm in federated learning, FedAvg updates the global model by performing a weighted average of local model parameters based on the proportion of data samples at each participating client, which is mathematically represented as:


$$\begin{array}{l} w_{t+1}=\overset{K}{\underset{k=1}{\sum }}\frac{n_{k}}{n}w_{t+1}^{k}, \end{array}$$ (1)

where *w*_*t*+1_ represents the updated global model parameters, *K* is the total number of clients, *n*_*k*_ is the data volume of the *k*-th client, *n* is the total data volume, and $w_{t+1}^{k}$ represents the local model parameters trained by the *k*-th client. He et al. (2021) propose a generalized framework for advanced visual tasks, FedCV, which serves as a benchmarking framework for high-level vision tasks. Wang et al. (2022) propose a privacy protection scheme for federated learning under edge computing by combining a lightweight masking protocol based on secret sharing, a digital signature algorithm, and a periodic average training strategy. Zhou et al. (2023) propose a participant selection method based on clustering and hierarchical federated learning, which utilizes social context data for group training and global aggregation of edge participants to achieve higher model accuracy and efficiency with fewer participants while ensuring data privacy. Despite FL's application in both low-level and high-level visual tasks, there currently lacks an FL framework tailored specifically for medical image fusion tasks. This paper proposes the FL-based method to medical image fusion.

#### 2.3.2 Personalized federated learning

Personalized federated learning (PFL) has emerged as a critical paradigm to tackle statistical data heterogeneity by customizing distributed models for individual local clients. Yang et al. (2023) propose a dynamic PFL method with adaptive differential privacy to address inflexible personalization. This method utilizes Fisher information to retain informative local parameters while mitigating the slow convergence caused by privacy clipping operations. Yang et al. (2024) present FedAS, a novel framework designed to bridge intra-client and inter-client inconsistencies in personalized federated learning. By leveraging federated parameter alignment and client synchronization, it effectively enhances local relevance and reduces the adverse impact of straggler clients. Chen et al. (2022) introduce pFL-Bench, a comprehensive benchmark tailored for evaluating diverse personalized federated learning methods. It provides a unified data partition and modularized codebase to facilitate reproducible and fair comparisons across different heterogeneous settings. Scott et al. (2024) propose PeFLL, a learning-to-learn approach that jointly trains an embedding network and a hypernetwork. This architecture maps clients into a latent descriptor space to output accurate personalized model parameters, especially in low-data regimes. Despite these significant advancements in general classification tasks, there are currently no personalized federated learning methods specifically designed to address the unique feature extraction and reconstruction challenges of multi-modal medical image fusion.

## 3 Method

This section presents the details of the proposed federated multi-modal medical image fusion framework. We first introduce the decoupled model architecture, and then elaborate on the comprehensive federated training scheme, which encompasses client-side model training, local evaluation, and server-side advanced aggregation.

### 3.1 Model architecture

**Figure 2:**
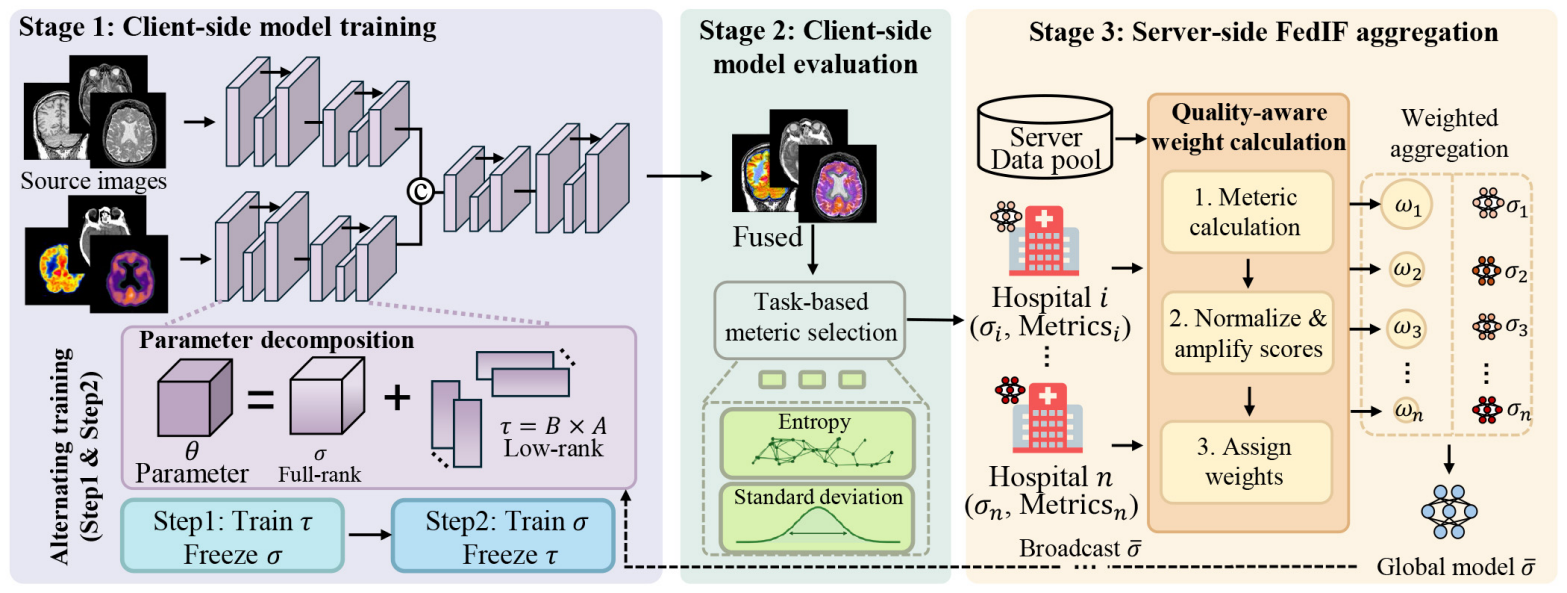
The architecture of FLMMIF.

The network architecture of the proposed model is illustrated in Figure 2. The model consists of three key components: a dual-branch modality-specific feature extraction layer, a channel attention-based feature fusion module, and a shared residual backbone network equipped with LoRA. To effectively capture the unique physiological and anatomical characteristics of different imaging modalities, we utilize a dual-branch input layer. Specifically, the input multi-modal images are processed by separate modality-specific convolutional layers, mapping each 1-channel input to a 32-channel feature map. In particular, these input layers are injected with LoRA modules to enhance local adaptability without incurring massive communication overhead.

Following independent feature extraction, modality-specific features are concatenated along the channel dimension. To emphasize the most informative features and suppress redundant information across modalities, we incorporate a Channel Attention mechanism after the concatenation and normalization steps. This attention-guided fusion ensures a high-quality intermediate representation.

The fused features are subsequently fed into a shared backbone composed of customized residual blocks. To balance the trade-off between global generalization and local personalization in a federated learning setting, we integrate LoRA into the convolutional layers of the residual blocks (controlled by the rank parameter *r*). This design allows the backbone to efficiently adapt to heterogeneous client data distributions by only updating the low-rank matrices during federated communication. Finally, an output head consisting of a single LoRA-injected convolutional layer is used to reconstruct the final fused image. Within the network structure, the convolutional kernels are uniformly designated as 3 × 3, accompanied by a padding of 1. Batch Normalization (BN) is utilized as the default normalization layer to stabilize the feature distribution and accelerate convergence. For the activation function, ReLU is the choice across all intermediate layers, while the final output layer utilizes Tanh to produce an output tensor ranging between [–1, 1].

### 3.2 Training scheme

#### 3.2.1 Client-side model training

To compensate for data heterogeneity across different medical centers and efficiently extract modality-specific features, we perform local model training in a distributed manner on the clients. For each client, the parameters of the local fusion model, denoted as Θ, are structurally decomposed into full-rank parameters σ and low-rank parameters τ. To reduce computational overhead while maintaining local adaptability, the low-rank component is parameterized as the product of two low-rank matrices, specifically τ = *B*×*A*.

As shown in Figure 2 stage 1, during the local training process, the client extracts multi-modal image pairs *I*_m1_ and *I*_m2_, from its local dataset, and feeds them into the network to reconstruct the fused image *I*_fused_. To effectively decouple the learning of personalized domain features from globally shared representations, we implement a two-step alternating optimization strategy for the local parameter updates:

##### 3.2.1.1 Train τ and freeze σ

In the first phase, we freeze the full-rank parameters σ and set only the low-rank parameters τ as trainable. This phase enables the model to rapidly adapt to the idiosyncratic data distribution and scanner-specific texture details of the individual client. Driven by the local fusion loss $L_{\text{fusion}}$, the update rule for τ at the local iteration *k* is formulated as:


$$\begin{array}{l} \tau^{k+1}=\tau^{k}-\eta \cdot \frac{\partial L_{\text{fusion}}}{\partial \tau^{k}}, \end{array}$$ (2)

where η denotes the learning rate.

##### 3.2.1.2 Train σ and freeze τ

Following the personalized tuning, we freeze the low-rank parameters τ and optimize the full-rank parameters σ. This second phase forces the full-rank backbone to learn generalized, domain-invariant fusion representations that are robust across different client modalities. The update rule for σ is defined as:


$$\begin{array}{l} \sigma^{k+1}=\sigma^{k}-\eta \cdot \frac{\partial L_{\text{fusion}}}{\partial \sigma^{k}}. \end{array}$$ (3)

By continuously alternating between these two steps during the local epochs, the client model dynamically balances its capacity to retain personalized anatomical details and acquire generalized fusion capabilities.

##### 3.2.1.3 Loss function

To guide the optimization of both the low-rank parameters τ and the full-rank parameters σ during the alternating local training, we design a comprehensive fusion loss function $L_{\text{fusion}}$. This total loss ensures that the fused image *I*_fused_ retains the optimal intensity distribution, structural integrity, and fine-grained texture gradients from the source multi-modal images (*I*_m1_ and *I*_m2_). The total fusion loss is defined as:


$$\begin{array}{l} L_{\text{fusion}}=\lambda_{1}L_{\text{int}}+\lambda_{2}L_{\text{grad}}+\lambda_{3}L_{\text{ssim}}, \end{array}$$ (4)

where λ_1_, λ_2_, and λ_3_ are weighting coefficients set to 2, 1, and 1, respectively, to balance the components.

To preserve the salient contrast and energy information from the source modalities, we utilize a weighted mean squared error (MSE). Based on the distinct imaging characteristics (e.g., MRI often providing superior soft-tissue contrast), the loss heavily anchors to the primary structural modality while preserving supplementary information:


$$\begin{array}{l} L_{\text{int}}=\text{MSE}\left(I_{\text{fused}},I_{\text{m2}}\right)+0.01\cdot \text{MSE}\left(I_{\text{fused}},I_{\text{m1}}\right). \end{array}$$ (5)

Texture details and edges are critical for medical diagnosis. We employ the Sobel operator ∇ to extract the gradient maps of the images. The gradient loss forces the fused image to match the maximum gradient values of the source images at each pixel location, computed via the *L*_1_-norm:


$$\begin{array}{l} L_{\text{grad}}=||\nabla I_{\text{fused}}-\max\left(\nabla I_{\text{m1}},\nabla I_{\text{m2}}\right){||}_{1}. \end{array}$$ (6)

To prevent structural distortion and maintain human-visual perception quality, we incorporate an SSIM-based penalty. We calculate a joint structural similarity that dynamically adapts to the standard deviations of local image patches across modalities:


$$\begin{array}{l} L_{\text{ssim}}=1-\text{SSIM}\left(I_{\text{fused}},I_{\text{m1}},I_{\text{m2}}\right). \end{array}$$ (7)

#### 3.2.2 Local evaluation

To mitigate the negative impact of low-quality local data or poorly converged models on the global shared backbone, we introduce a performance-based local evaluation mechanism. In standard federated learning (e.g., FedAvg), client models are typically aggregated based solely on their local data volume. However, in medical image fusion, the complexity of the data and the absence of a single ground truth necessitate a quality-driven assessment. Therefore, after completing the local alternating training phase, each client *j* evaluates its updated model on a local validation set before transmitting any parameters to the server. Because fusion tasks cannot be evaluated using simple classification accuracy, we construct a comprehensive evaluation score *S*_*j*_ relying on objective image quality metrics and the local optimization loss.

As shown in Figure 2 stage 2, we extract two key statistical indicators from the generated fusion images: *(i)* Information Entropy (*EN*): Measures the richness of the structural and texture information successfully transferred from the source modalities into the fused image. *(ii)* Standard Deviation (*SD*): Assesses the overall pixel intensity distribution, reflecting the global contrast of the fused image. To compute the final evaluation score, we reward models that produce high-contrast, information-rich images while strictly penalizing those with a high residual fusion loss. The evaluation score *S*_*j*_ for the *j*-th client is mathematically formulated as:


$$\begin{array}{l} S_{j}=\frac{EN+SD}{L_{\text{fusion}}+\epsilon }, \end{array}$$ (8)

where $L_{\text{fusion}}$ is the total loss computed during the validation forward pass, and ϵ = 10^−6^ is a remarkably small constant added to the denominator to prevent division by zero and ensure numerical stability. EN measures the amount of information contained in the fused image, defined as:


$$\begin{array}{l} \text{EN}=-\overset{L-1}{\underset{l=0}{\sum }}p_{l}\log_{2}p_{l}, \end{array}$$ (9)

where *L* is the total number of gray levels, and *p*_*l*_ is the probability of a pixel having the gray level *l* in *I*_fused_. A higher EN indicates richer texture details and information. SD reflects the contrast and pixel distribution dispersion of the image, defined as:


$$\begin{array}{l} \text{SD}=\sqrt{\frac{1}{MN}\overset{M}{\underset{i=1}{\sum }}\overset{N}{\underset{j=1}{\sum }}{\left(I_{\text{fused}}\left(i,j\right)-\mu \right)}^{2}}, \end{array}$$ (10)

where *M* and *N* denote the spatial dimensions (height and width) of the fused image, *I*_fused_(*i, j*) is the pixel value at coordinates (*i, j*), and μ is the mean pixel value of the entire image.

A higher *S*_*j*_ indicates that the client's local model has successfully converged and is capable of generating high-quality fused images with preserved anatomical structures and distinct contrast. Once computed, this scalar score *S*_*j*_, alongside the shared full-rank parameters σ, is uploaded to the central server to guide the dynamic weighting in the subsequent aggregation phase.

#### 3.2.3 Server-side FedIF aggregation

In standard federated learning, the server aggregates local models by assigning weights strictly based on the size of the clients' local datasets. However, in medical image fusion scenarios, data quality and task difficulty vary significantly across different scanners and centers. To address this, as shown in Figure 2 stage 3, we propose a performance-based aggregation strategy on the server side, FedIF, which dynamically adjusts the aggregation weights based on the local evaluation scores.

During each communication round *t*, after the local training is completed, the server collects the full-rank parameters $\sigma_{i}^{t}$ and the evaluation score *S*_*i*_ from each participating client *i*. To ensure numerical stability and prevent exponential explosion, the raw scores are first standardized using Z-score normalization:


$$\begin{array}{l} {Ŝ}_{i}=\frac{S_{i}-\mu_{S}}{\sigma_{S}+\epsilon }, \end{array}$$ (11)

where μ_*S*_ and σ_*S*_ denote the mean and standard deviation of the scores across all participating clients in the current round, and ϵ is a small constant. Subsequently, a Softmax function with a temperature scaling mechanism is applied to map the normalized scores into dynamic aggregation weights. The weight *w*_*i*_ for the *i*-th client is calculated as:


$$\begin{array}{l} w_{i}=\frac{\exp\left({Ŝ}_{i}/T\right)}{\sum_{j=1}^{K}\exp\left({Ŝ}_{j}/T\right)}, \end{array}$$ (12)

where *K* is the total number of participating clients, and *T* = 0.5 is the temperature hyperparameter controlling the sensitivity of the weights to the score differences. A smaller *T* amplifies the influence of high-performing clients.

Crucially, to preserve the personalized capabilities learned by each client, the server performs a selective parameter aggregation. As designed in our decoupled model architecture, the locally trained low-rank parameters τ are strictly kept local and never uploaded to the server. The server only performs weighted aggregation on the shared full-rank backbone parameters σ:


$$\begin{array}{l} \sigma_{\text{global}}^{t}=\overset{K}{\underset{i=1}{\sum }}w_{i}\sigma_{i}^{t}. \end{array}$$ (13)

Finally, the updated generalized global parameters $\sigma_{\text{global}}^{t}$ are broadcast back to all clients to replace their local full-rank parameters, preparing the system for the next round of alternating local optimization. This advanced aggregation ensures that the global backbone benefits most from clients generating high-quality fusions, while entirely preventing the loss of personalized detail extraction capabilities.

## 4 Experiments

In this section, we first introduce the experimental setup, including datasets, training detail, and comparison methods. Later, we present qualitative and quantitative results on three datasets.

### 4.1 Setting up

#### 4.1.1 Datasets

We train FLMMIF on the public *Harvard* medical image dataset for three distinct multimodal fusion tasks and test its subjective and objective performance across these modalities. The training and test datasets include CT-MRI, PET-MRI, and SPECT-MRI image pairs, all sourced entirely from the *Harvard* dataset. Specifically, for the CT-MRI fusion task, we select 160 pairs of CT and MRI images as the training set, and 24 pairs of images as the test set. For the PET-MRI fusion task, we select 245 pairs of PET and MRI images as the training set, and choose 24 pairs of images as the test set. For the SPECT-MRI fusion task, we select 333 pairs of SPECT and MRI images as the training set, and choose 24 pairs of images as the test set.

**Figure 3:**
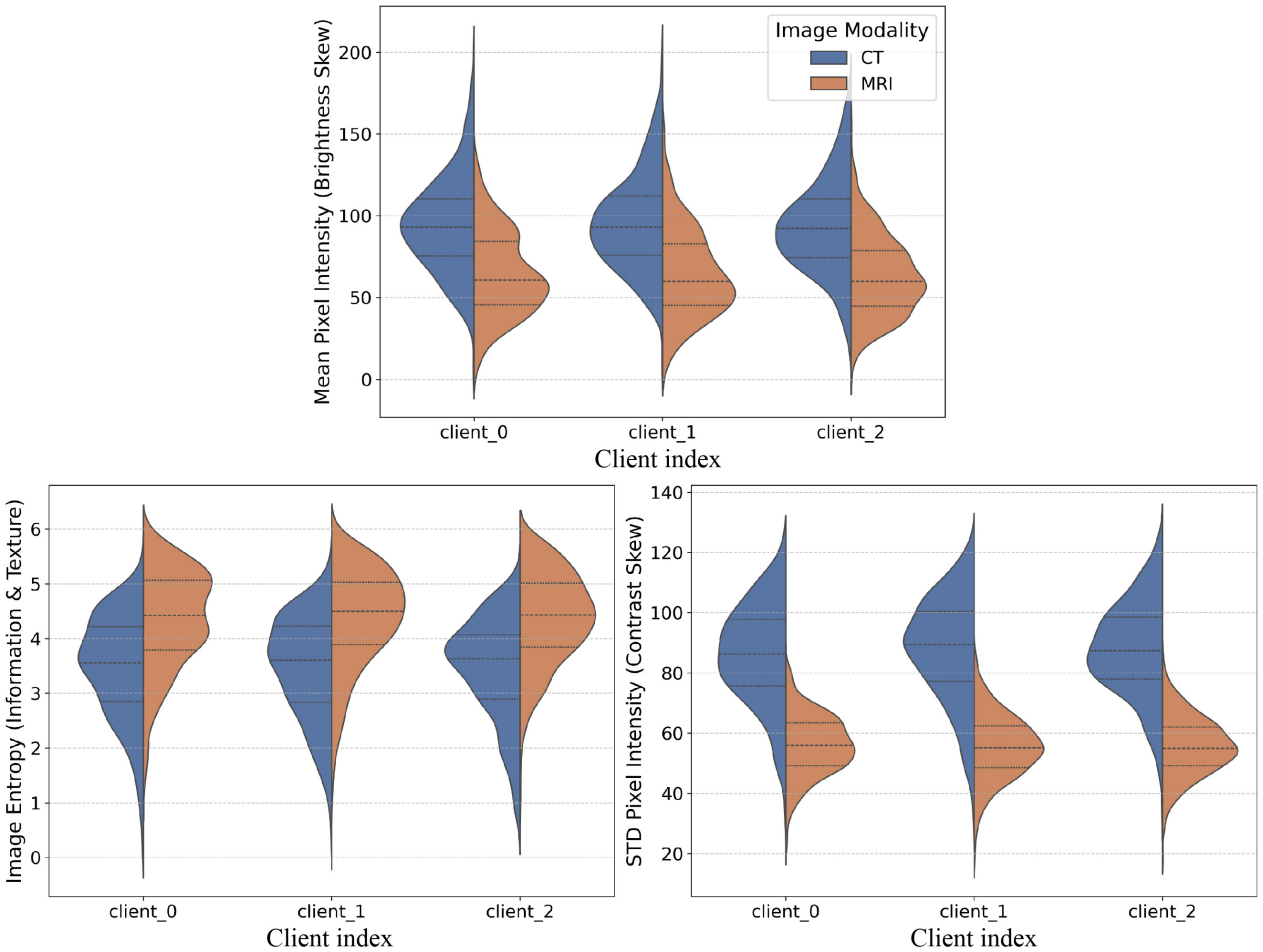
Data distribution differences of CT and MRI source images across clients.

The data for the same fusion task is evenly distributed among the clients within the federated network. However, to simulate a realistic scenario, the data exhibits a non-IID property in terms of feature representation. As illustrated in Figure 3, the statistical characteristics of the source images—including mean pixel intensity, standard deviation, and image entropy—display noticeable variations and feature skew across different clients for both CT and MRI modalities. For example, in FLMMIF with 20 clients, each client contains 8 training image pairs for the CT-MRI task, 12 pairs for the PET-MRI task, and 27 pairs for the SPECT-MRI task. Note that we crop the training images into patches using a sliding window method to augment the dataset.

#### 4.1.2 Training detail

Our experiments are conducted on an NVIDIA RTX 4090 GPU during training and testing. In the experiments, we configure a total of 200 communication rounds for the federated training process. A total of 3 clients participate in the system, and all clients are selected in each round (the fraction of clients is set to 1). During the local training phase of each round, clients perform 3 local epochs to update the shared model parameters, followed by 2 local epochs specifically dedicated to updating the personalized part of the network.

For the model architecture, we utilize ResNet-10 as the backbone to construct a dual-branch encoder integrated with a channel attention mechanism. We optimize the network parameters using the SGD optimizer with a momentum of 0.9. The initial learning rate is set at 0.01. The local training batch size is set to 50, and the test batch size is set to 10. Additionally, for the parameter decomposition settings, the rank configuration for the convolutional layers is set to 0.6, and the rank for the fully connected layers is set to 10.

#### 4.1.3 Comparison methods

We compare FLMMIF with seven state-of-the-art medical image fusion methods, including CSMCA (Zhang and Feng, 2025), MMIF-INet (He et al., 2025), RCGAN (Li et al., 2019), U2Fusion (Xu et al., 2020), EMMA (Zhao et al., 2024), GeSeNet (Li et al., 2023), and MATR (Tang et al., 2022). Additionally, we establish baselines to evaluate the effectiveness of our federated architecture and the specific components. We compare against a standard FedAvg (Kairouz and McMahan, 2021) implementation and a No-LoRA variant. We aggregate the shared backbone models deployed on the clients based on standard FedAvg to demonstrate the limitations of conventional data-volume-weighted aggregation. The No-LoRA variant removes the low-rank matrices from the network, which we use as a representative baseline to validate the necessity of parameter-efficient fine-tuning for local personalization. Note that during the client-side training phase, we adopt LoRA as the local adapter to capture specific modal features, and the detailed ablation performance of different strategies and loss functions is discussed in Section IV.E. We employ metrics including structural similarity (SSIM), peak signal-to-noise ratio (PSNR), multi-scale structural similarity (MS-SSIM), visual quality metric (*Q*_CB_), learned perceptual image patch similarity (LPIPS), and deep image structure and texture similarity (DISTS) to objectively evaluate the quality of the fusion results.

### 4.2 Quantitative results

Tables 1–3 quantitatively present the comparison results between our proposed FLMMIF and seven state-of-the-art fusion methods on the CT-MRI, PET-MRI, and SPECT-MRI datasets.

**Table 1:** Quantitative comparison on CT-MRI datasets.

Methods	SSIM ↑	PSNR ↑	MS-SSIM ↑	*Q*_*CB*_ ↑	LPIPS ↓	DISTS ↓
CSMCA	**0.5910**	15.2257	**0.9187**	**0.6526**	**0.1663**	**0.2064**
MMIF-INet	0.5775	14.4336	0.9158	0.6200	0.2111	0.2454
RCGAN	0.2216	**15.5111**	0.8254	0.3060	0.1798	0.2279
U2Fusion	0.2356	15.4729	0.8409	0.3201	0.1875	0.2412
EMMA	0.4783	14.6692	**0.9192**	0.5557	0.1749	0.2173
GeSeNet	0.2509	14.3145	0.9170	0.3673	0.1779	0.2108
MATR	0.2269	**18.4519**	0.5809	0.3571	**0.1618**	**0.2018**
FLMMIF	**0.6362**	15.3359	0.7730	**0.6616**	0.1979	0.2209

The red color represents the best performance, and the blue color represents the second-best performance. ↑ denotes higher values are better, and ↓ denotes lower values are better.

Overall, across the 18 evaluation metrics on the three datasets, FLMMIF achieved 6 first-place and 9 second-place results. These results demonstrate that our method can generate fusion results that preserve complete texture structures, provide better visual perception, and exhibit lower noise under the federated learning paradigm, while strictly protecting user privacy.

Specifically, for the CT-MRI dataset shown in Table 1, FLMMIF achieves the best value in SSIM (0.6362), and *Q*_*CB*_ (0.6616). CT imaging excels at visualizing dense tissues such as bones and calcifications, while MRI offers superior contrast for soft tissues. The optimal SSIM indicates that FLMMIF perfectly preserves the rigid, high-frequency bone structures from the CT images and the fine soft-tissue boundaries from the MRI images. Additionally, the *Q*_*CB*_ values demonstrate that the fused images maintain excellent perceptual quality with minimal artifact interference, successfully avoiding the blurring of anatomical details often caused by standard federated aggregation methods.

In the PET-MRI fusion task shown in Table 2, FLMMIF also achieves excellent results, obtaining the best value in DISTS (0.2663) and the second-best values across all other metrics. PET images are highly sensitive to functional metabolic activity but lack spatial resolution, whereas MRI provides precise structural information. The comparable metrics indicate that our method can effectively integrate the functional metabolic color information of PET into the high-resolution MRI anatomical structures without causing spatial distortion. Furthermore, the lowest DISTS value proves that the structural and textural similarities to the source images are highly preserved, ensuring that lesions or active metabolic areas can be accurately localized within the soft-tissue background.

**Table 2:** Quantitative comparison on PET-MRI datasets.

Methods	SSIM ↑	PSNR ↑	MS-SSIM ↑	*Q*_*CB*_ ↑	LPIPS ↓	DISTS ↓
CSMCA	**0.5870**	15.4342	0.9310	**0.6082**	0.3406	0.2885
MMIF-INet	0.5522	15.1985	**0.9502**	0.5725	0.3469	0.2885
RCGAN	0.2744	16.0421	0.8213	0.3813	0.3559	0.2910
U2Fusion	0.2674	15.2494	0.8463	0.3606	0.3532	0.2998
EMMA	0.3575	14.8969	0.9318	0.4907	0.3492	0.2811
GeSeNet	0.3348	15.9598	0.9267	0.4077	0.3487	0.2920
MATR	0.3017	**17.3425**	0.7965	0.3643	**0.3181**	**0.2671**
FLMMIF	**0.5698**	**16.4381**	**0.9320**	**0.5796**	**0.3363**	**0.2663**

The red color represents the best performance, and the blue color represents the second-best performance. ↑ denotes higher values are better, and ↓ denotes lower values are better.

Finally, as shown in Table 3, FLMMIF demonstrates outstanding performance on the SPECT-MRI dataset; specifically, its SSIM, PSNR, LPIPS, and DISTS reach 0.5907, 21.7879, 0.2568, and 0.2395, respectively. Similar to PET, SPECT provides crucial functional perfusion information. The significant improvements in LPIPS and DISTS compared to the sub-optimal methods show that FLMMIF can seamlessly integrate low-resolution functional signals with the high-resolution structural details of MRI. This ensures that the generated fusion results not only retain complete textures and minimize noise but also align closely with human visual perception, achieving an optimal balance between functional highlighting and anatomical structure preservation while strictly protecting user privacy.

**Table 3:** Quantitative comparison on SPECT-MRI dataset.

Methods	SSIM ↑	PSNR ↑	MS-SSIM ↑	*Q*_*CB*_ ↑	LPIPS ↓	DISTS ↓
CSMCA	**0.5620**	20.9158	0.9465	**0.6452**	0.2744	0.2783
MMIF-INet	0.5582	18.6496	**0.9690**	0.6089	0.2784	0.2832
RCGAN	0.2338	20.3499	0.8859	0.3722	0.2770	0.2704
U2Fusion	0.2459	19.5298	0.8912	0.3375	0.2781	0.2820
EMMA	0.3820	18.5877	0.9501	0.5033	0.2755	0.2684
GeSeNet	0.3049	20.5653	0.9582	0.4018	0.2791	0.2815
MATR	0.2567	**21.1156**	0.8890	0.3568	**0.2569**	**0.2456**
FLMMIF	**0.5907**	**21.7879**	**0.9584**	**0.6362**	**0.2568**	**0.2395**

The red color represents the best performance, and the blue color represents the second-best performance. ↑ denotes higher values are better, and ↓ denotes lower values are better.

### 4.3 Qualitative results

**Figure 4:**
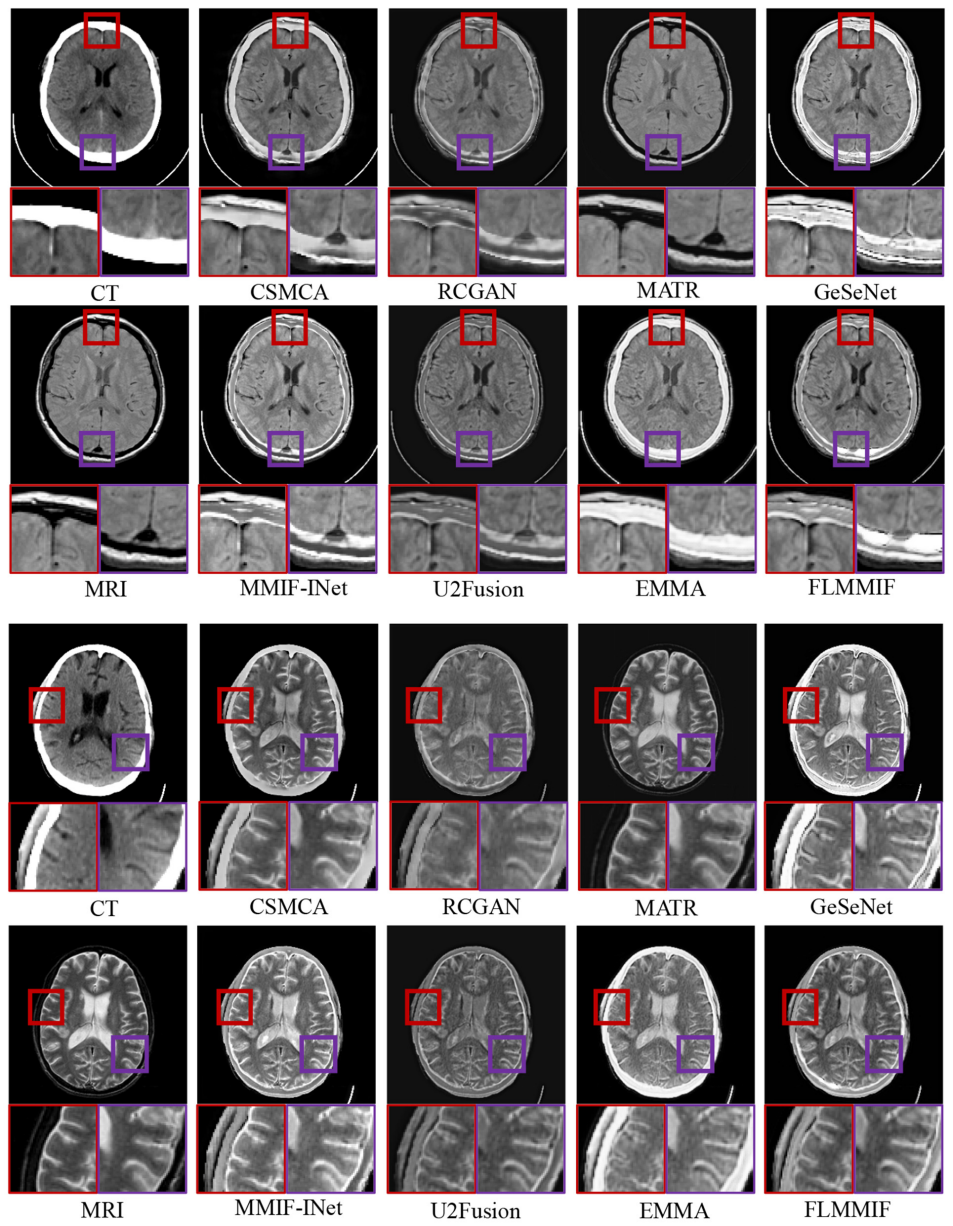
Qualitative assessment on CT-MRI dataset.

**Figure 5:**
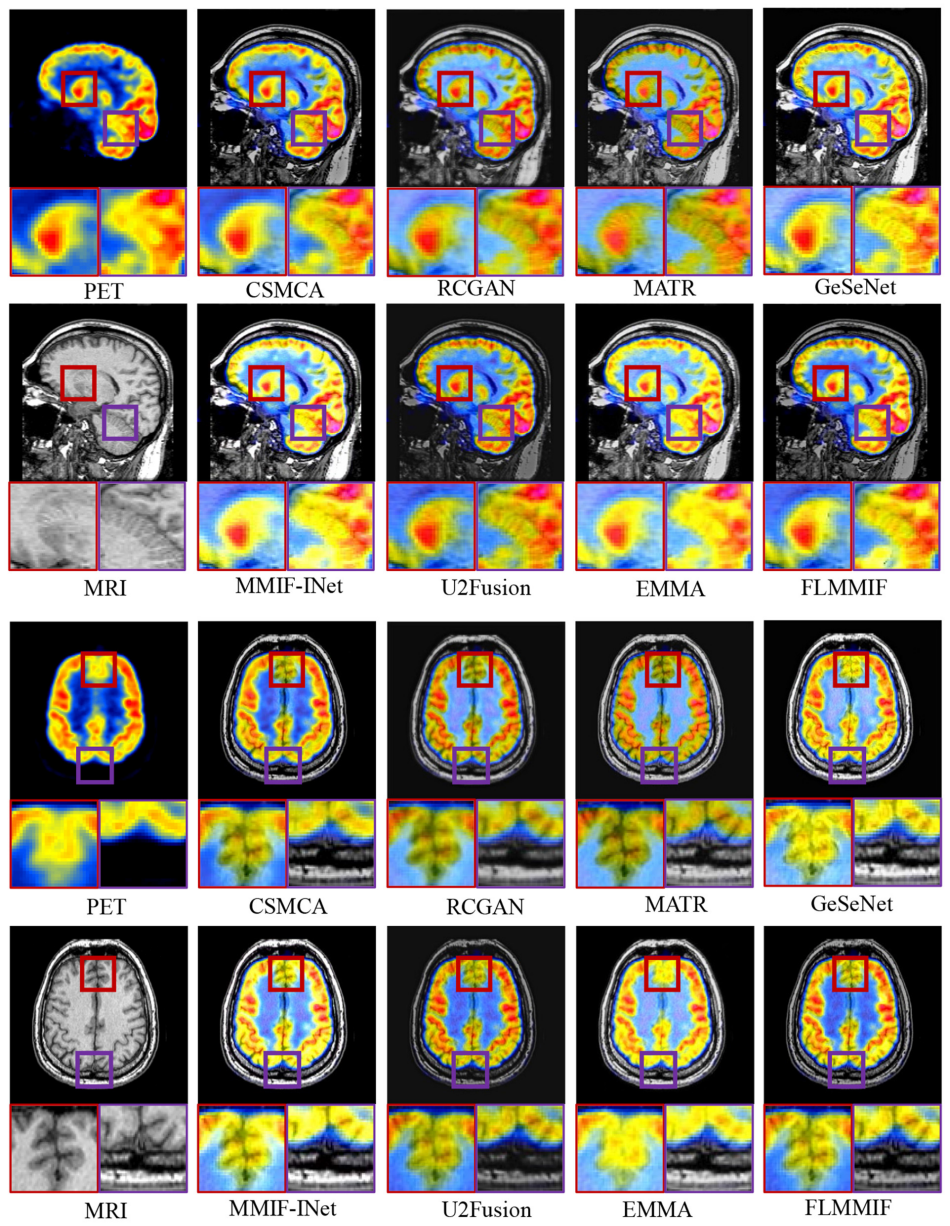
Qualitative assessment on PET-MRI dataset.

**Figure 6:**
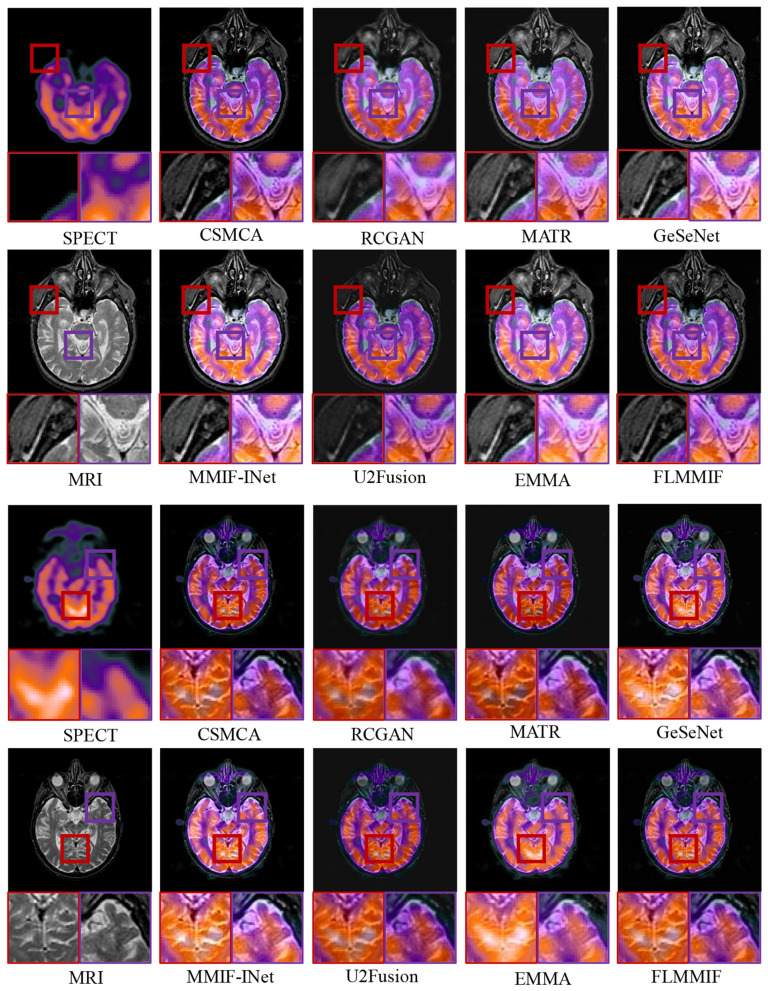
Qualitative assessment on SPECT-MRI dataset.

This section conducts a visual quality comparison of the fused images generated by different methods. Figures 4–6 present the qualitative fusion results of our proposed FLMMIF and other comparison methods on the CT-MRI, PET-MRI, and SPECT-MRI datasets, respectively.

Specifically, as shown in Figure 4, FLMMIF demonstrates superior visual performance when fusing CT and MRI images. CT imaging primarily provides dense structures such as bone and skull boundaries, while MRI captures clear soft-tissue details. Compared to existing centralized methods which often suffer from contrast reduction or structural blurring, FLMMIF maintains the sharp, high-frequency bone boundaries of CT and the rich soft-tissue textures of MRI. Crucially, this high-quality visual preservation is achieved within a secure Federated Learning environment, indicating that our method does not sacrifice image clarity or anatomical details to protect data privacy.

Furthermore, Figure 5 illustrates the qualitative results for the PET-MRI dataset. PET images highlight functional and metabolic activities through color distribution but lack anatomical precision, whereas MRI provides a high-resolution structural background. As shown in the magnified regions, several existing methods fail to maintain the color fidelity of PET signals or introduce significant spatial distortion into the MRI background. In contrast, FLMMIF effectively injects functional metabolic color information into the structural background. The fused images exhibit vibrant functional highlights and clear anatomical boundaries, confirming the robust feature extraction capability of our decoupled federated network without centralizing sensitive patient data.

Finally, Figure 6 presents the visual fusion results for the SPECT-MRI dataset. SPECT images provide vital functional perfusion information. Visual comparisons reveal that the fusion results generated by FLMMIF possess the highest perceptual quality, with reduced noise and unnatural artifacts. The low-resolution functional regions from SPECT are accurately mapped onto the high-resolution soft-tissue areas of the MRI. This intuitively demonstrates that our alternating local training and targeted server-side aggregation strategy enable the model to perfectly balance functional highlighting and anatomical preservation across different medical centers, producing clinically valuable images while keeping all raw data strictly localized.

### 4.4 Parameters

**Figure 7:**
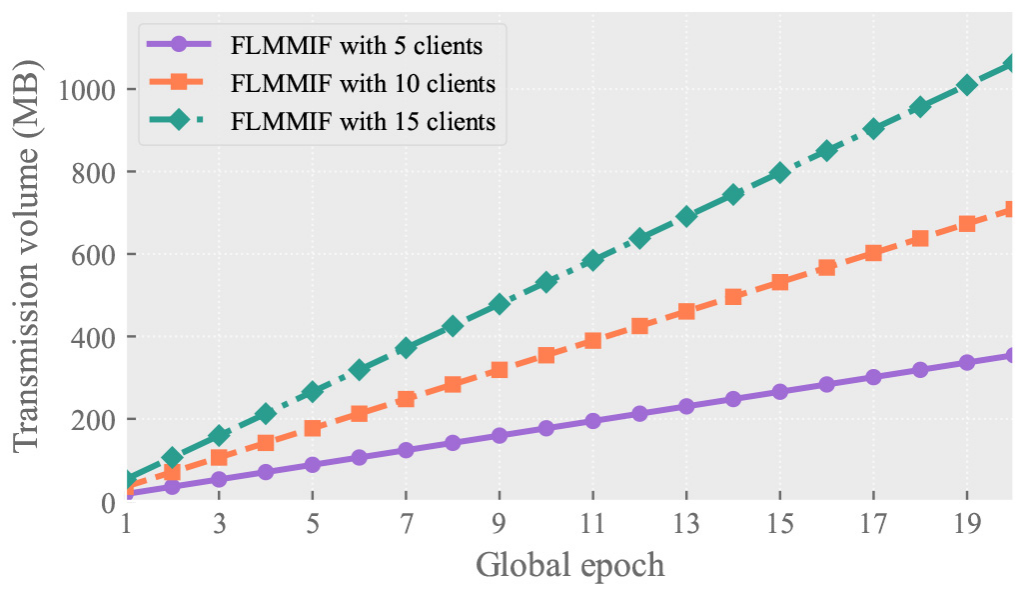
FLMMIF communication parameter analysis.

**Figure 8:**
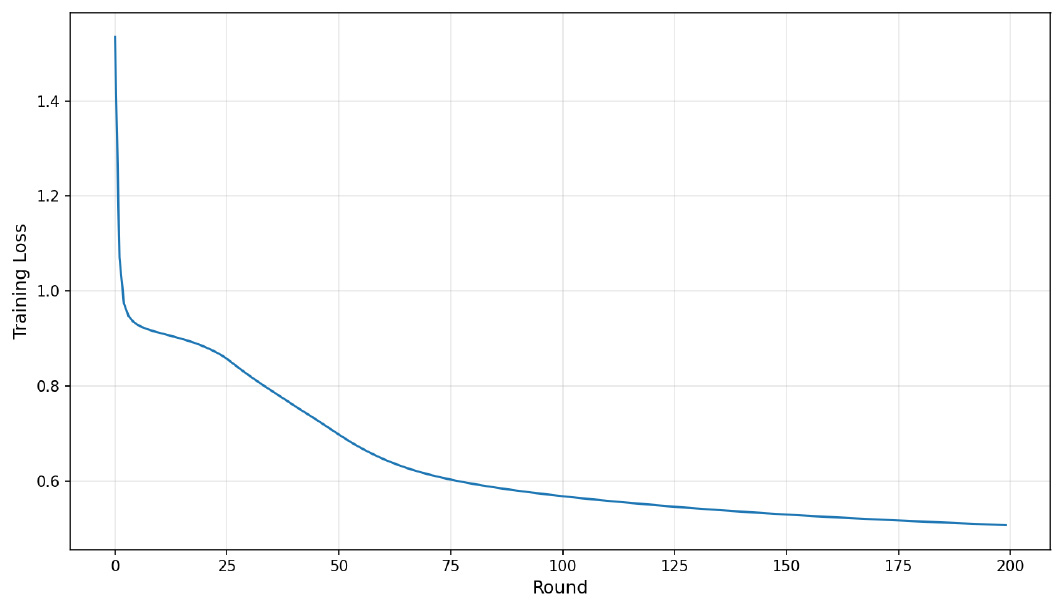
Training loss convergence over rounds.

To evaluate the communication efficiency of the proposed FLMMIF framework in a practical federated learning environment, we conduct a quantitative analysis of the parameter transmission volume during the training process. As shown in the Figure 7, we record the cumulative communication transmission volume generated as the global training epochs increase under the participation of different numbers of clients (5, 10, and 15). Furthermore, the training loss convergence curve is illustrated in Figure 8. As depicted, the training loss drops rapidly during the initial communication rounds and gradually converges to a stable value around 0.5 after 200 rounds, demonstrating the stable and effective convergence behavior of the proposed FLMMIF framework.

At the end of the 20th round, the total transmission volume for 5 clients is approximately 354.34 MB, the total transmission volume for 10 clients is approximately 708.67 MB, and the total transmission volume for 15 clients is approximately 1,063.01 MB. For any given number of clients, the cumulative data transmission volume exhibits a strictly linear growth trend as the number of global epochs increases. This indicates that the size of the parameters transmitted in each round of communication aggregation is fixed and highly controllable, without an exponential inflation of the communication burden. During the training process, although the clients alternately optimize the full-rank and low-rank parameters locally, they only upload and download the shared full-rank backbone network parameters when communicating with the server. These results demonstrate that FLMMIF not only excels in fusion performance but also possesses extremely high deployment feasibility and scalability in real-world Internet of Medical Things scenarios with bandwidth constraints.

### 4.5 Ablation study

#### 4.5.1 Effectiveness of the loss functions

To verify the effectiveness of each component in the proposed loss function, we conducted quantitative and qualitative ablation studies on three datasets: CT-MRI, PET-MRI, and SPECT-MRI. Table 4 presents the quantitative comparison results. In Table 4, w/o $L_{grad}$ indicates the removal of the gradient loss term during the training process, w/o $L_{ssim}$ denotes the removal of the structural similarity loss term, and FLMMIF (Full) represents the complete proposed method incorporating all loss components. As shown in Table 4, when any single loss component is removed, the model's performance experiences varying degrees of decline across all six evaluation metrics: SSIM, PSNR, MS-SSIM, *Q*_*CB*_, LPIPS, and DISTS. Conversely, the complete FLMMIF method maintains optimal performance levels across all metrics on the three datasets. These results demonstrate the effectiveness of the fusion loss, which is capable of guiding the distributed fusion network to generate fusion results with complete texture structures and human-visual perception quality in a data-secure environment.

**Table 4:** Ablation study on loss functions (CT-MRI, PET-MRI, and SPECT-MRI datasets).

Dataset	Method	SSIM ↑	PSNR ↑	MS-SSIM ↑	*Q*_*CB*_ ↑	LPIPS ↓	DISTS ↓
CT-MRI	w/o $L_{grad}$	0.6286	13.7824	0.6215	0.6592	0.2137	0.2344
	w/o $L_{ssim}$	0.5887	14.9515	0.6124	0.6364	0.2154	0.2300
	**FLMMIF (Full)**	**0.6362**	**15.3359**	**0.7730**	**0.6616**	**0.1979**	**0.2209**
PET-MRI	w/o $L_{grad}$	0.3409	14.1686	0.6290	0.4910	0.3603	0.3022
	w/o $L_{ssim}$	0.3845	**16.6915**	0.7702	0.3973	0.3370	0.2790
	**FLMMIF (Full)**	**0.5698**	16.4381	**0.9320**	**0.5796**	**0.3363**	**0.2663**
SPECT-MRI	w/o $L_{grad}$	0.5338	19.8261	0.7046	0.5827	0.2676	0.2573
	w/o $L_{ssim}$	0.4962	21.7769	0.8811	0.4842	0.2667	0.2596
	**FLMMIF (Full)**	**0.5907**	**21.7879**	**0.9584**	**0.6362**	**0.2568**	**0.2395**

↑ denotes higher is better, ↓ denotes lower is better. Bold values indicate the optimal performance under each evaluation metric.

**Figure 9:**
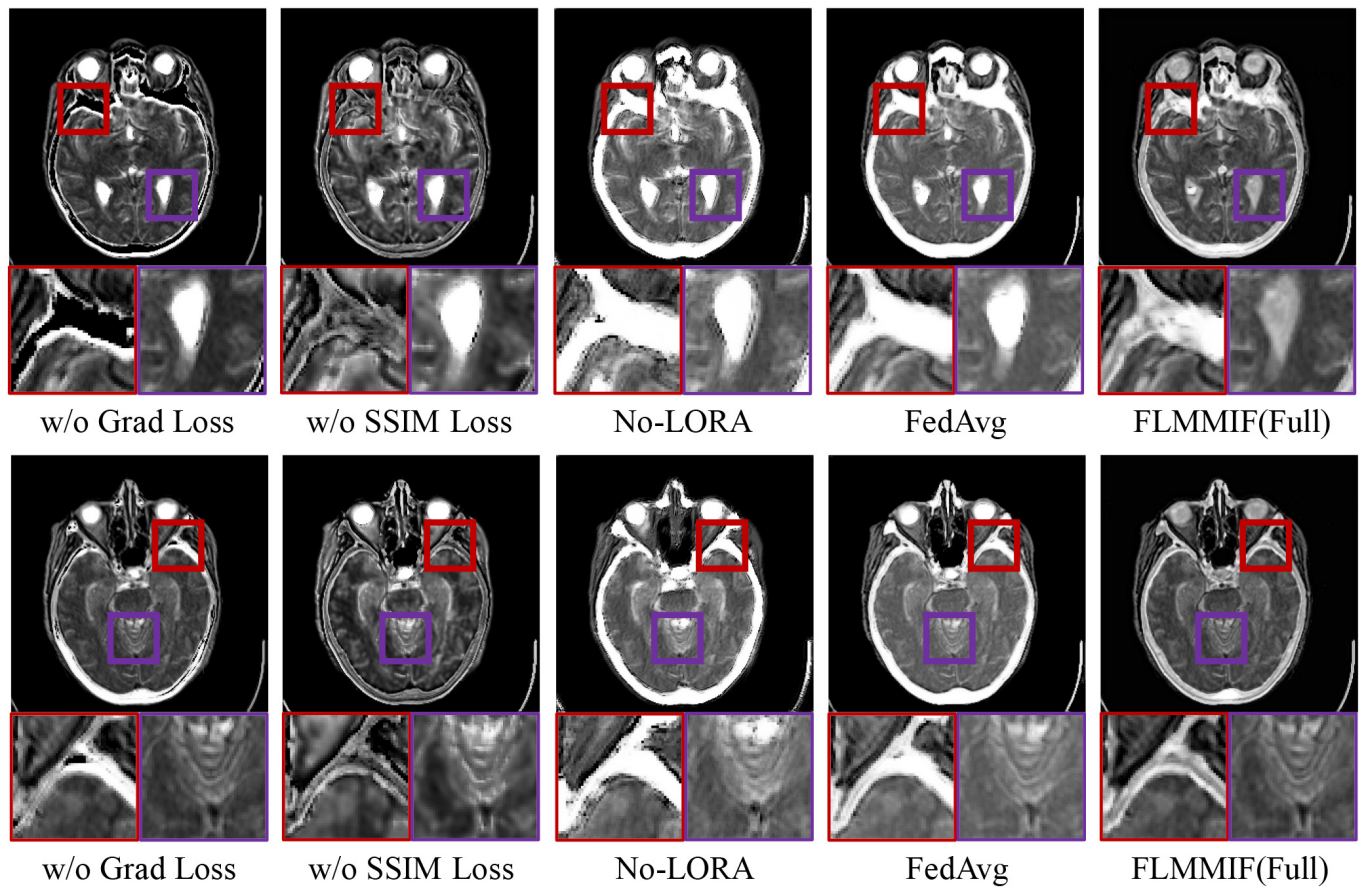
Quantitative assessment of ablation study on CT-MRI dataset.

**Figure 10:**
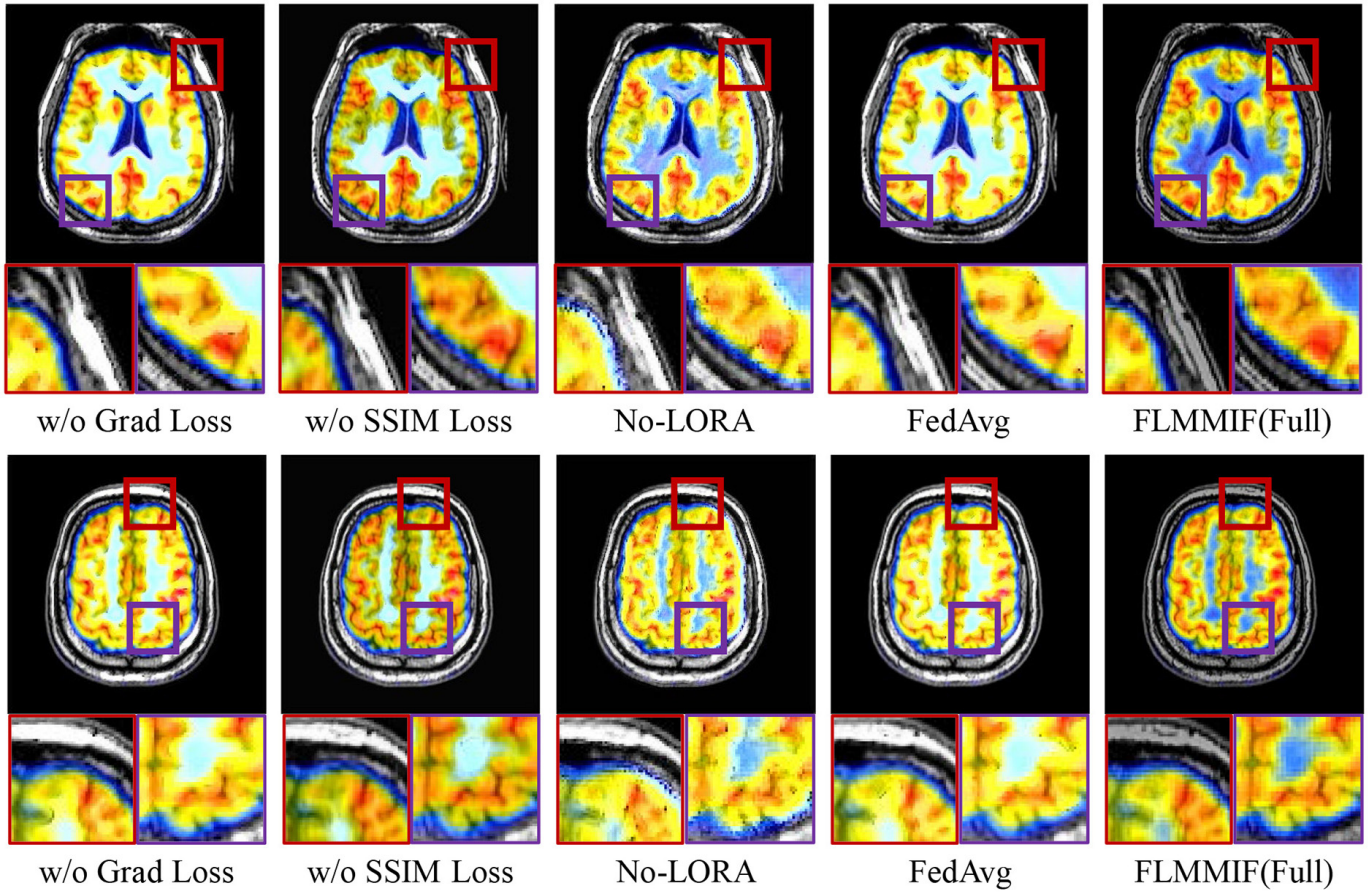
Quantitative assessment of ablation study on PET-MRI dataset.

**Figure 11:**
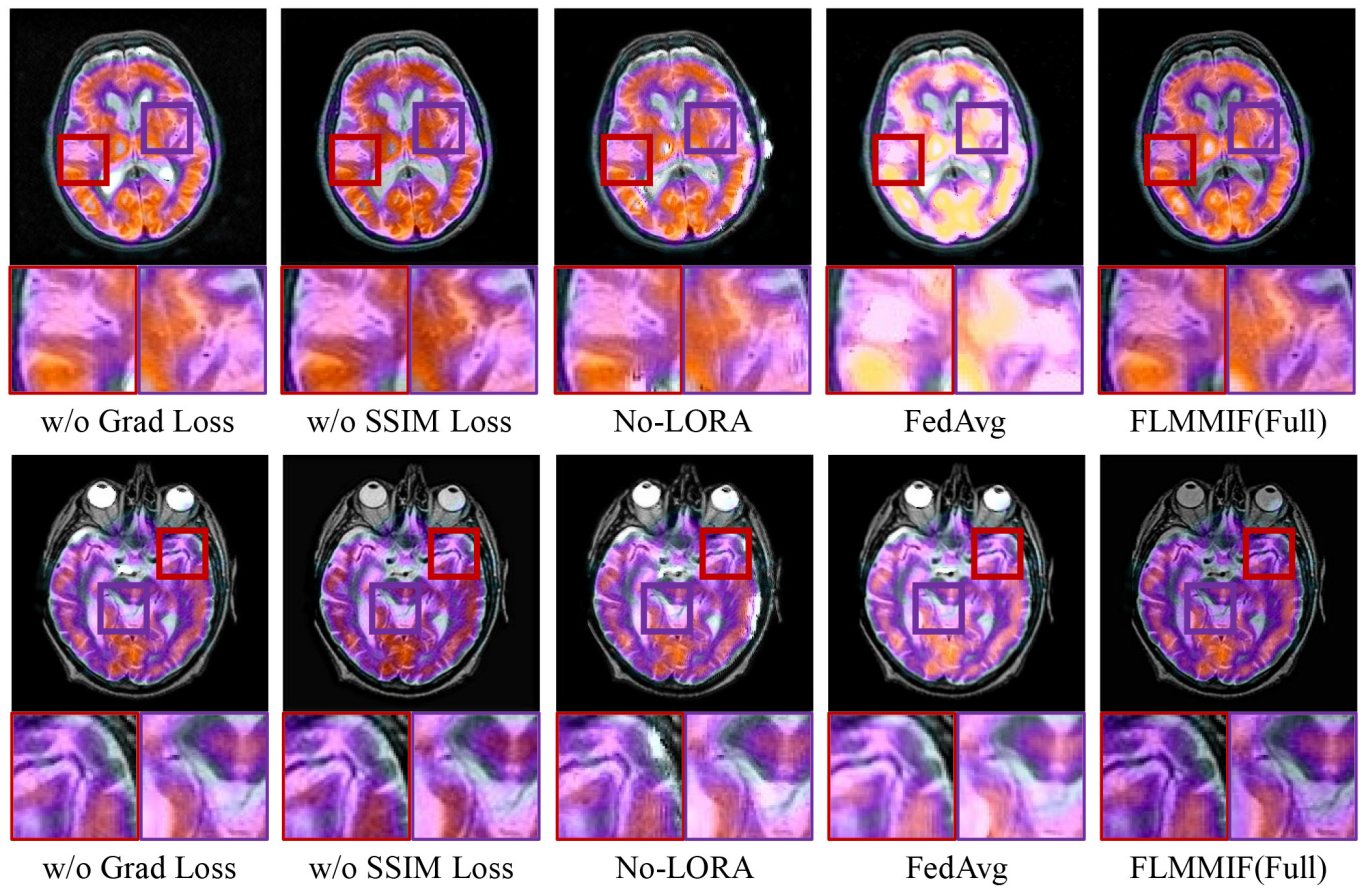
Quantitative assessment of ablation study on SPECT-MRI dataset.

The first two columns of Figures 9–11 display the qualitative visual ablation results regarding the loss functions on the CT, PET, and SPECT datasets, respectively. The primary role of the gradient loss $L_{grad}$ is to force the fused image to match the maximum gradient values of the source images at each pixel location, thereby extracting critical texture details and edges. From the visual results, it can be observed that when $L_{grad}$ is removed (w/o Grad Loss), the generated fused images exhibit distinct edge blurring and lose the sharp anatomical boundaries present in the source images. Furthermore, the SSIM-based penalty term $L_{ssim}$ is introduced to prevent structural distortion and maintain good human-visual perception quality. In the images where $L_{ssim}$ is removed (w/o SSIM Loss), the overall contrast and structural coherence are compromised, leading to a decrease in visual fidelity. In comparison, the FMMIF (Full) method employing the complete loss function not only preserves complete, sharp high-frequency texture details and clear anatomical structures, but also maintains high contrast and natural visual perception quality, demonstrating the best comprehensive fusion performance.

#### 4.5.2 Effectiveness of personalized fine-tuning

To verify the necessity of parameter-efficient fine-tuning for achieving local personalization, we design the No-LORA baseline experiment. In this setting, the network removes all low-rank matrices and trains using only the globally shared backbone network.

As Table 5 shows, the performance of the No-LORA model drops significantly on the three datasets CT-MRI, PET-MRI, and SPECT-MRI. For example, in the PET-MRI dataset, the SSIM metric of No-LORA plummets to 0.2593, and the PSNR is only 13.8180. This is because under the federated learning environment, data from different medical institutions often exhibit significant statistical heterogeneity, and they possess unique feature distributions that a single global model cannot perfectly accommodate. Lacking the local fine-tuning of the LoRA mechanism, a single global model struggles to adapt to the unique modality features and scanner-specific texture details of each client.

**Table 5:** Comparison with different strategies (No-LoRA and FedAvg) across three datasets.

Dataset	Method	SSIM ↑	PSNR ↑	MS-SSIM ↑	*Q*_*CB*_ ↑	LPIPS ↓	DISTS ↓
CT-MRI	No-LoRA	0.5507	11.9563	0.5827	0.6155	0.2641	0.2592
	FedAvg	**0.6417**	13.5114	0.6146	0.6385	**0.1946**	**0.2179**
	**FLMMIF (Full)**	0.6362	**15.3359**	**0.7730**	**0.6616**	0.1979	0.2209
PET-MRI	No-LoRA	0.2593	13.8180	0.6651	0.4313	0.3756	0.2961
	FedAvg	0.5566	13.8412	0.6636	0.5827	0.3535	0.2933
	**FLMMIF (Full)**	**0.5698**	**16.4381**	**0.9320**	**0.5796**	**0.3363**	**0.2663**
SPECT-MRI	No-LoRA	0.3987	17.2606	0.6773	0.5499	0.3075	0.2782
	FedAvg	0.5487	14.2347	0.6798	0.6025	0.3121	0.2917
	**FLMMIF (Full)**	**0.5907**	**21.7879**	**0.9584**	**0.6362**	**0.2568**	**0.2395**

The best results are highlighted in bold. ↑ denotes higher values are better, and ↓ denotes lower values are better.

Nevertheless, as shown in Table 5, FedAvg marginally outperforms FLMMIF (Full) on the CT-MRI dataset in terms of SSIM, LPIPS, and DISTS. This trade-off stems from the unique modality characteristics: unlike PET/SPECT-MRI tasks that integrate functional signals into structural templates, CT-MRI is a dual-high-resolution structural fusion task. Under such mild heterogeneity, the forced parameter averaging in standard FedAvg acts as a strong global regularizer, yielding a smooth structural consensus that aligns slightly better with the global test set. In contrast, FLMMIF utilizes local low-rank matrices to capture client-specific scanner variations. While this precise personalization induces a negligible drop in strict global alignment for CT-MRI, it effectively prevents the severe structural blurring and artifacts caused by FedAvg in highly heterogeneous non-IID scenarios (e.g., PET/SPECT-MRI), thereby maintaining significant overall superiority.

As the qualitative visual results show in Figures 9–11, the fused images generated by No-LORA perform poorly in preserving specific modality features, exhibit reduced overall contrast, and fail to preserve the details of the source images well. Conversely, the complete FLMMIF framework successfully allows the model to dynamically coordinate specific local features with general global knowledge by decoupling the global full-rank parameters and local low-rank parameters, thereby achieving local personalization without sacrificing the capabilities of the backbone network.

#### 4.5.3 Effectiveness of FedIF

In FedAvg, the server typically allocates model aggregation weights solely based on the size of the local data volume of the clients. To verify the advantages of our proposed performance metric based aggregation strategy FedIF, we compare FedIF with the baseline based on the standard FedAvg algorithm.

As the quantitative results in Table 5 show, compared to conventional FedAvg, FLMMIF Full achieves significant improvements across the vast majority of objective evaluation metrics. Especially in key metrics reflecting image fidelity and structural similarity such as PSNR and MS-SSIM, the advantage of FLMMIF is particularly obvious. For instance, in the SPECT-MRI dataset, the PSNR of FLMMIF is 21.7879, while that of FedAvg is only 14.2347; the MS-SSIM of FLMMIF is 0.9584, and that of FedAvg is only 0.6798. This significant performance gap proves that the traditional data volume based weight allocation fails to handle the uneven data quality and task difficulty across different nodes in medical image fusion tasks. Our proposed strategy effectively avoids the negative impact of low quality local data or unconverged models on the globally shared backbone network by calculating image quality based evaluation scores on the client side and dynamically adjusting the aggregation weights accordingly on the server side.

As the visual comparisons in Figures 9–11 show, models aggregated using FedAvg often introduce blurring or insufficient contrast in their generated images due to the forced averaging of heterogeneous data. In contrast, the fusion results of FLMMIF Full not only perfectly preserve high resolution anatomical structures but also effectively and clearly integrate functional color signals. This proves that this advanced aggregation mechanism maximizes the absorption of advantages from high quality nodes, thereby comprehensively improving the fusion performance of the global model.

#### 4.5.4 Hyperparameter sensitivity analysis

**Figure 12:**
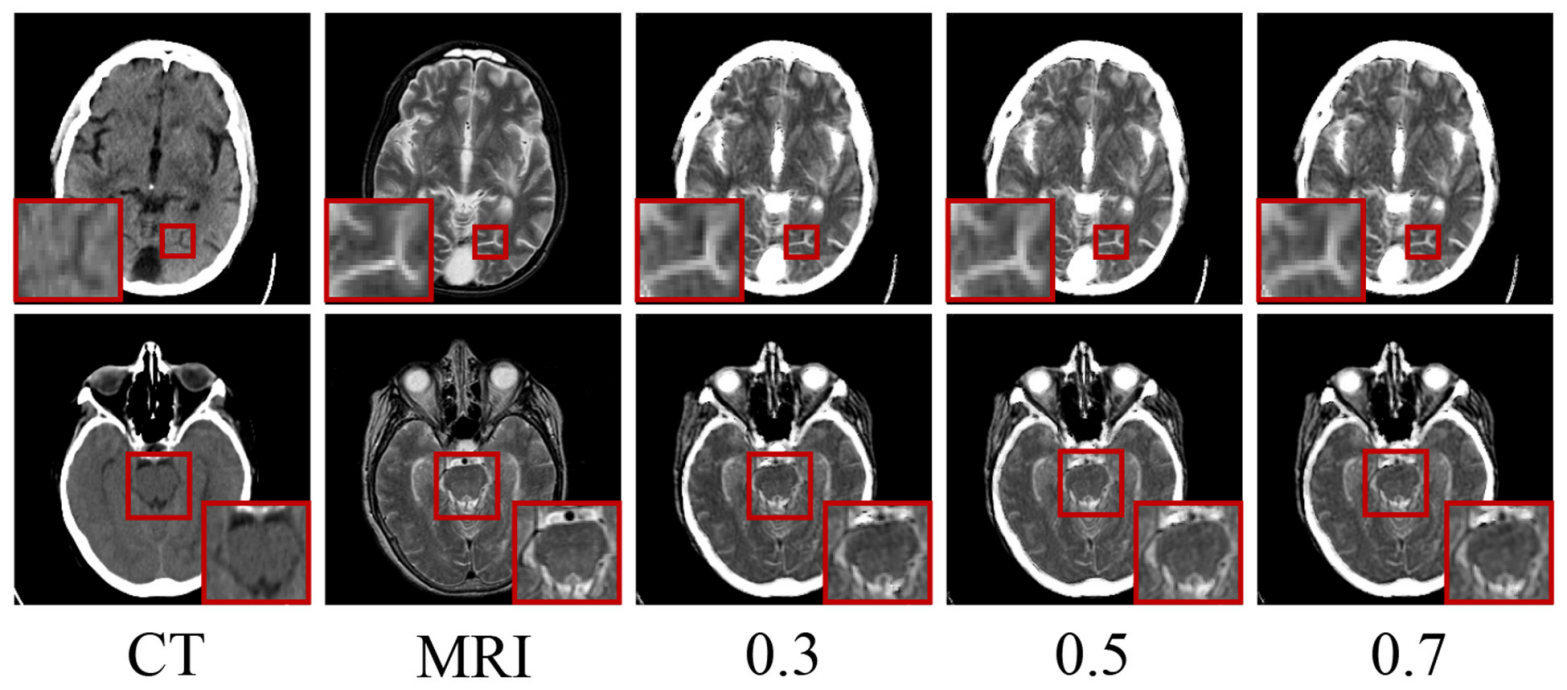
Ablation study of *T*.

To ensure that the dynamic allocation of aggregation weights optimizes the fusion performance of the global model, we added an ablation study on the temperature hyperparameter T in the Softmax function. For fairness, we set T to 0.3, 0.5, and 0.7, respectively, during the same training process to alter the sensitivity of the weights to score differences. The qualitative and quantitative results are shown in Figure 12 and Table 6, respectively.

**Table 6:** Sensitivity analysis of the temperature parameter *T* used in the server-side softmax function.

Method	SSIM ↑	PSNR ↑	MS-SSIM ↑	*Q*_*CB*_ ↑	LPIPS ↓	DISTS ↓
*T* = 0.3	**0.7078**	**13.5388**	0.6027	**0.4535**	0.2049	0.2261
*T* = 0.5	**0.7078**	13.4584	**0.6028**	0.4521	**0.2037**	0.2245
*T* = 0.7	0.7052	13.5030	0.6023	0.4512	0.2056	**0.2244**

Bold values indicate the optimal performance under each evaluation metric. ↑ denotes higher values are better, and ↓ denotes lower values are better.

The qualitative results are illustrated in Figure 12. Overall, when T is 0.5, the fusion results exhibit the best visual perception quality and structural consistency. For example, as can be seen from the magnified regions in the figure, when T = 0.3 or T = 0.7, the fused images are slightly inadequate in preserving details at the edges of soft tissues, and the contrast is somewhat reduced. However, when T = 0.5, the image not only perfectly preserves the high-frequency bone boundaries from the CT image but also accurately integrates the rich soft-tissue texture details from the MRI image. These results demonstrate that when T = 0.5, the server can most reasonably allocate the aggregation weights, enabling FLMMIF to effectively protect data privacy while dynamically coordinating specific local features with general global knowledge. This generates high-quality multi-modal fused images that are highly reliable for clinical decision-making.

The quantitative results are presented in Table 6. Overall, when T = 0.5, the model achieves the optimal comprehensive performance across various objective evaluation metrics. Specifically, although the PSNR (13.5388) and QCB (0.4535) metrics are slightly higher when T = 0.3, the model at T = 0.5 not only maintains an equally excellent SSIM (0.7078) and achieves the highest MS-SSIM (0.6028), but also reaches the lowest optimal value (0.2037) in the LPIPS metric, which measures perceptual image patch similarity. Because a lower LPIPS indicates that the generated fused image aligns more closely with human visual perception in terms of deep structure and texture features , combining the above objective data leads to the conclusion that setting T to 0.5 enables the FedIF mechanism to maximize the absorption of advantages from high-quality nodes. Consequently, this comprehensively and robustly enhances the medical image fusion performance of the global model within a federated learning framework that strictly adheres to privacy protection.

**Figure 13:**
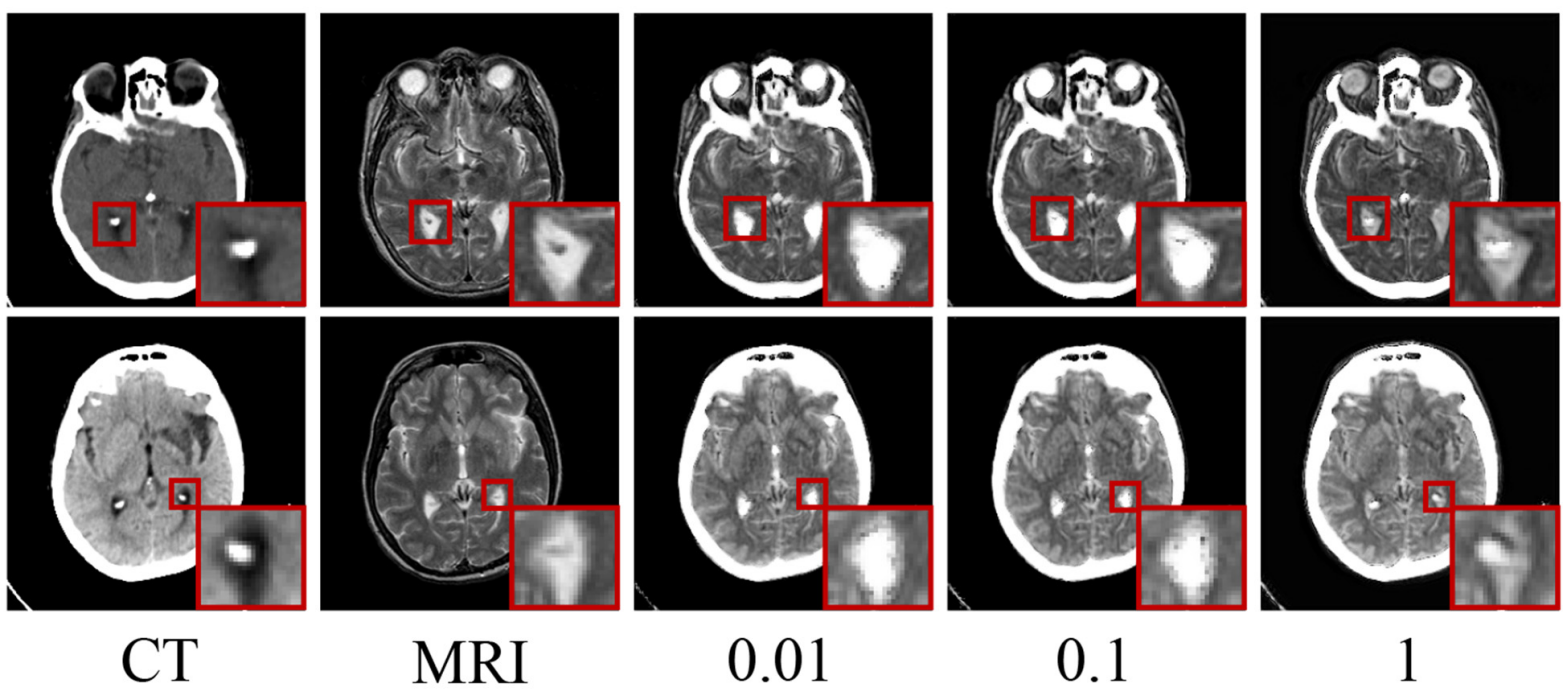
Ablation study of the weighting coefficient in $L_{\text{int}}$.

To ensure that a reasonable balance of the multi-modal intensity loss $L_{\text{int}}$ optimizes the fusion performance of the global model, we have added a sensitivity analysis section regarding its weighting coefficient. The qualitative and quantitative results are shown in Figure 13 and Table 7, respectively.

**Table 7:** Sensitivity analysis of the weighting coefficient in $L_{\text{int}}$.

Method	SSIM ↑	PSNR ↑	MS-SSIM ↑	*Q*_*CB*_ ↑	LPIPS ↓	DISTS ↓
1	**0.7078**	13.4584	0.6028	0.4521	0.2037	0.2245
0.1	0.7087	13.5806	0.6042	0.4564	0.2010	0.2227
0.01	0.6362	**15.3359**	**0.7730**	**0.6616**	**0.1979**	**0.2209**

Bold values indicate the optimal performance under each evaluation metric. ↑denotes higher values are better, and ↓denotes lower values are better.

The qualitative results are illustrated in Figure 13. Overall, when the default setting of FLMMIF is adopted, the fusion results exhibit the best visual perceptual quality and structural consistency. For example, as can be seen from the magnified local regions in the figure, when the weighting coefficient is set to 0.1 or 1, the fused images are slightly deficient in the energy integration of different modal features and fail to perfectly balance their brightness and contrast, leading to a minor degradation in the boundary details of certain anatomical structures. Conversely, when the default setting of FLMMIF is employed, the image not only perfectly preserves the high-frequency bone and cranial boundaries from the CT image but also accurately integrates the rich soft-tissue texture details from the MRI image. These results indicate that when the 0.01 weighting coefficient of FLMMIF is adopted, the system can most reasonably guide the joint distribution of intensities, enabling FLMMIF to effectively protect data privacy while dynamically coordinating specific local features with general global knowledge, thereby generating high-quality multi-modal fused images with high reliability for clinical decision-making.

The quantitative results are presented in Table 7. Overall, when the weighting coefficient is set to 0.01, the model achieves the optimal comprehensive performance across various objective evaluation metrics. Specifically, although the SSIM metrics are slightly higher when the weighting coefficient is 1 or 0.1 (0.7078 and 0.7087, respectively), the model with the 0.01 setting achieves a significantly higher PSNR (15.3359), the highest MS-SSIM (0.7730), and the highest *Q*_*CB*_ (0.6616). Furthermore, it reaches the lowest optimal values in both the LPIPS metric (0.1979), which measures perceptual image patch similarity, and the DISTS metric (0.2209), which evaluates deep image structure and texture similarity. A higher PSNR and MS-SSIM signify superior image structural fidelity, whereas a lower LPIPS and DISTS imply that the generated fused images align more closely with human visual perception in terms of deep structures and texture features. These results demonstrate that setting the weighting coefficient to 0.01 enables the loss function to most effectively guide the parameter optimization of the distributed fusion network, thereby comprehensively and robustly enhancing the medical image fusion performance of the global model within a federated learning framework that strictly adheres to privacy protection.

#### 4.5.5 Effectiveness of aggregation methods

**Figure 14:**
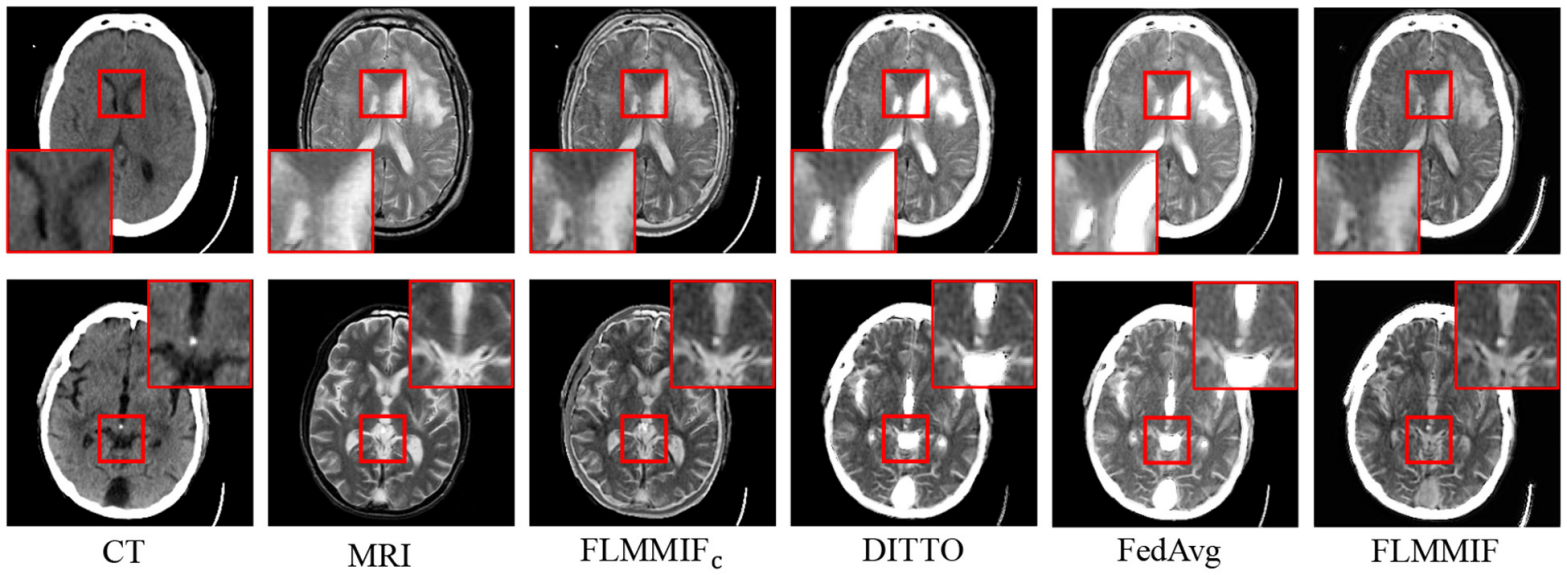
Comparison between the proposed FLMMIF, the FedAvg baseline.

To evaluate the effectiveness of the proposed federated aggregation method in optimizing the fusion performance of the global model, we added a comparative study against standard baselines and a theoretical upper bound. The qualitative and quantitative results are shown in Figure 14 and Table 8, respectively. In this evaluation, FLMMIF_*c*_ represents the centralized oracle version, where all data is centrally aggregated without privacy constraints.

**Table 8:** Quantitative comparison between the proposed FLMMIF, the FedAvg baseline, and the centralized oracle version.

Method	SSIM ↑	PSNR ↑	MS-SSIM ↑	*Q*_*CB*_ ↑	LPIPS ↓	DISTS ↓
FedAvg	0.7066	13.6225	0.6013	0.4523	0.2033	0.2233
Ditto	0.7081	13.6336	0.6028	0.4511	0.2024	0.2227
FLMMIF	0.6362	15.3359	**0.7730**	**0.6616**	0.1979	0.2209
FLMMIF_*c*_	**0.7219**	**15.4084**	0.6230	0.5112	**0.1867**	**0.2100**

Bold values represent the best results, and underline values indicate the second-best results. ↑ denotes higher values are better, and ↓denotes lower values are better.

The qualitative results are illustrated in Figure 14. Overall, the fusion results of the proposed FLMMIF exhibit a visual perceptual quality and structural consistency that are highly comparable to the centralized oracle, FLMMIF_*c*_. For example, as can be seen from the magnified local regions in the figure, distributed baseline methods like DITTO are slightly deficient in perfectly balancing brightness and contrast, leading to a minor degradation in the boundary details of certain anatomical structures when compared to the centralized model. Conversely, the FLMMIF image not only effectively preserves the high-frequency cranial boundaries from the CT image but also accurately integrates the rich soft-tissue texture details from the MRI image, visually approaching the optimal performance of FLMMIF_*c*_. These results indicate that the proposed aggregation mechanism can dynamically coordinate specific local features with general global knowledge, thereby generating high-quality multi-modal fused images without requiring centralized access to raw, sensitive patient data.

The quantitative results are presented in Table 8. Overall, FLMMIF_*c*_ serves as the theoretical upper bound, achieving optimal values in metrics such as SSIM (0.7219), PSNR (15.4084), LPIPS (0.1867), and DISTS (0.2100). However, among the privacy-preserving distributed methods, FLMMIF achieves the optimal comprehensive performance. Specifically, FLMMIF achieves a significantly higher MS-SSIM (0.7730) and the highest *Q*_*CB*_ (0.6616) compared to FedAvg and Ditto. Furthermore, its LPIPS (0.1979) and DISTS (0.2209) metrics closely trail the centralized oracle and consistently outperform the distributed baselines. A higher MS-SSIM signifies superior image structural fidelity, while a lower LPIPS indicates that the generated fused image aligns more closely with human visual perception. Consequently, this comprehensively and robustly enhances the medical image fusion performance of the global model to approach centralized levels within a federated learning framework that strictly adheres to privacy protection.

#### 4.5.6 Client quantity analysis

**Figure 15:**
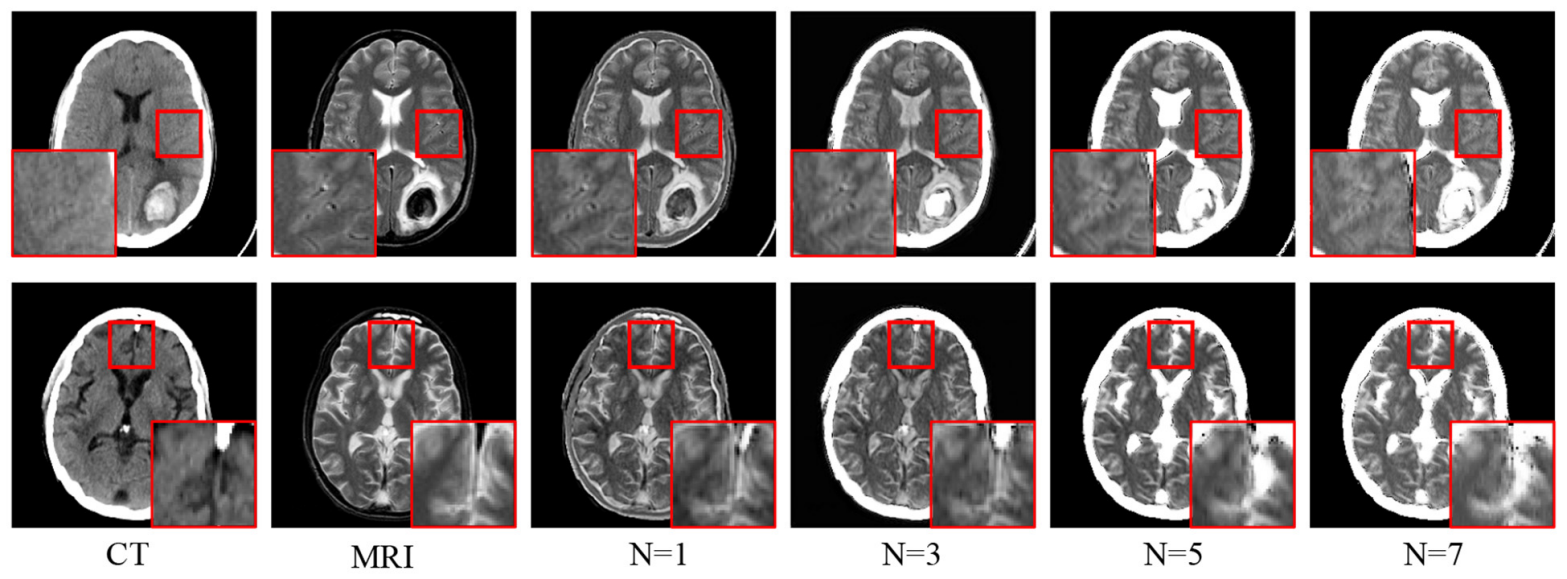
Ablation study of clinet number.

To investigate the impact of the number of participating clients on the fusion performance and scalability of our federated framework, we conducted a client quantity analysis. The qualitative and quantitative results for different numbers of clients (*N* = 1 for the centralized oracle, *N* = 3, *N* = 5, and *N* = 7) are presented in Figure 15 and Table 9, respectively. In this evaluation, FLMMIF_*c*_ represents the centralized theoretical upper bound where all data is aggregated and trained without privacy constraints.

**Table 9:** Performance comparison under different numbers of clients.

Method	SSIM ↑	PSNR ↑	MS-SSIM ↑	*Q*_*CB*_ ↑	LPIPS ↓	DISTS ↓
FLMMIF_7_	0.6488	10.8825	0.5334	0.4254	0.2970	0.2744
FLMMIF_5_	0.6729	12.5440	0.5798	0.4331	0.2502	0.2477
FLMMIF_3_	0.6362	15.3359	**0.7730**	**0.6616**	0.1979	0.2209
FLMMIF_*c*_	**0.7219**	**15.4084**	0.6230	0.5112	**0.1867**	**0.2100**

Bold values represent the best results, and underline values indicate the second-best results. ↑ denotes higher values are better, and ↓ denotes lower values are better.

The qualitative visual results are illustrated in Figure 15. As observed in the magnified local regions of the CT-MRI fusion task, the visual quality remains relatively stable and robust across different client settings. When *N* = 3, the generated fused images effectively preserve the high-frequency structural details, such as the cranial boundaries from the CT images, and the clear soft-tissue textures from the MRI images, achieving visual fidelity closely resembling the centralized model (*N* = 1). As the network scales to include more distributed clients (*N* = 5 and *N* = 7), although a slight reduction in contrast and minor edge smoothing can be observed, the fused images consistently preserve the essential anatomical structures without introducing severe artifacts. This indicates that the framework successfully maintains its fundamental fusion capabilities, producing clinically acceptable and highly readable results even when integrating increasingly distributed and heterogeneous data.

The quantitative results in Table 9 further corroborate this robustness. While there is a predictable and manageable fluctuation in certain metrics as the client pool expands, the overall performance remains within an acceptable and highly reliable range. Among the distributed configurations, FLMMIF_3_ achieves the optimal comprehensive performance, reaching an MS-SSIM of 0.7730 and a PSNR of 15.3359. As the number of clients increases to 5 and 7, the models continue to deliver dependable results. For instance, FLMMIF_7_ maintains an SSIM of 0.6488, which is comparable to the 0.6362 achieved by FLMMIF_3_, demonstrating that the core structural similarity is exceptionally well-preserved at scale. While certain metrics such as PSNR (12.5440 for FLMMIF_5_ and 10.8825 for FLMMIF_7_) and MS-SSIM experience a moderate decrease, this reduction is a standard, bearable, and highly acceptable trade-off given the exponentially increased statistical heterogeneity of multi-center non-IID data. Crucially, our decoupled LoRA mechanism and FedIF aggregation strategy effectively constrain these performance variations, ensuring that the performance reduction is minimal and that the shared global backbone retains a strong generalization capacity.

## 5 Discussion and limitations

While FLMMIF prevents direct data leakage through architectural data-locality, standard privacy attacks (e.g., gradient inversion) and defenses (e.g., Differential Privacy) are primarily targeted at supervised learning and are largely inapplicable to unsupervised medical image fusion. Unsupervised loss functions do not explicitly encode input-label mappings for attackers to exploit, and applying DP noise would severely degrade the high-frequency structural details essential for clinical diagnosis.

Regarding clinical deployment, a primary limitation is the reliance on perfectly registered image pairs. Real-world spatial misalignments can introduce ghosting artifacts during fusion. Furthermore, while our LoRA strategy handles moderate scanner variations, extreme heterogeneity—such as missing sequences or severe motion artifacts—remains challenging. Future research will focus on integrating federated cross-modal registration and adaptive mechanisms to address these real-world complexities.

## 6 Conclusion

This paper introduces FLMMIF, a privacy-preserving framework integrating a federated learning paradigm and low-rank adaptation for personalized and secure medical image fusion. We observe that existing centralized methods typically raise serious data privacy concerns, while standard distributed approaches often fail to balance global generalization with local node personalization due to data heterogeneity. To address this, we propose a two-stage iterative strategy that ensures the model dynamically coordinates specific local features with general global knowledge before parameter transmission. The local training phase utilizes a dual-branch encoder and single-branch decoder, initially training low-rank parameters to secure local personalization, followed by training full-rank parts to guarantee global baseline performance. Subsequently, a metric-based aggregation mechanism FedIF is established on the server, which evaluates the performance of uploaded models to assign higher aggregation weights to superior nodes for optimized global updating. Extensive experiments demonstrate that FLMMIF generates high-quality fusion results across three distinct multimodal fusion tasks, effectively protecting data privacy while keeping the size of transmitted parameters fixed and highly controllable during communication.

## Data Availability

The original contributions presented in the study are included in the article/supplementary material, further inquiries can be directed to the corresponding author.
